# RPPA SPACE: an R package for normalization and quantitation of Reverse-Phase Protein Array data

**DOI:** 10.1093/bioinformatics/btac665

**Published:** 2022-10-07

**Authors:** Huma Shehwana, Shwetha V Kumar, James M Melott, Mary A Rohrdanz, Chris Wakefield, Zhenlin Ju, Doris R Siwak, Yiling Lu, Bradley M Broom, John N Weinstein, Gordon B Mills, Rehan Akbani

**Affiliations:** Department of Bioinformatics and Computational Biology, The University of Texas MD Anderson Cancer Center, Houston, TX 77030, USA; Department of Bioinformatics and Computational Biology, The University of Texas MD Anderson Cancer Center, Houston, TX 77030, USA; Department of Bioinformatics and Computational Biology, The University of Texas MD Anderson Cancer Center, Houston, TX 77030, USA; Department of Bioinformatics and Computational Biology, The University of Texas MD Anderson Cancer Center, Houston, TX 77030, USA; Department of Bioinformatics and Computational Biology, The University of Texas MD Anderson Cancer Center, Houston, TX 77030, USA; Department of Bioinformatics and Computational Biology, The University of Texas MD Anderson Cancer Center, Houston, TX 77030, USA; Department of Genomic Medicine, The University of Texas MD Anderson Cancer Center, Houston, TX 77030, USA; Department of Genomic Medicine, The University of Texas MD Anderson Cancer Center, Houston, TX 77030, USA; Department of Bioinformatics and Computational Biology, The University of Texas MD Anderson Cancer Center, Houston, TX 77030, USA; Department of Bioinformatics and Computational Biology, The University of Texas MD Anderson Cancer Center, Houston, TX 77030, USA; Department of Systems Biology, The University of Texas MD Anderson Cancer Center, Houston, TX 77030, USA; Division of Oncological Sciences, Knight Cancer Institute, Oregon Health and Science Center, Portland, OR 97210, USA; Department of Bioinformatics and Computational Biology, The University of Texas MD Anderson Cancer Center, Houston, TX 77030, USA

## Abstract

**Summary:**

Reverse-Phase Protein Array (RPPA) is a robust high-throughput, cost-effective platform for quantitatively measuring proteins in biological specimens. However, converting raw RPPA data into normalized, analysis-ready data remains a challenging task. Here, we present the RPPA SPACE (RPPA *S*uperposition *A*nalysis and *C*oncentration *E*valuation) R package, a substantially improved successor to SuperCurve, to meet that challenge. SuperCurve has been used to normalize over 170 000 samples to date. RPPA SPACE allows exclusion of poor-quality samples from the normalization process to improve the quality of the remaining samples. It also features a novel quality-control metric, ‘noise’, that estimates the level of random errors present in each RPPA slide. The noise metric can help to determine the quality and reliability of the data. In addition, RPPA SPACE has simpler input requirements and is more flexible than SuperCurve, it is much faster with greatly improved error reporting.

**Availability and implementation:**

The standalone RPPA SPACE R package, tutorials and sample data are available via https://rppa.space/, CRAN (https://cran.r-project.org/web/packages/RPPASPACE/index.html) and GitHub (https://github.com/MD-Anderson-Bioinformatics/RPPASPACE).

**Supplementary information:**

Supplementary data are available at *Bioinformatics* online.

## 1 Introduction

The Reverse-Phase Protein array (RPPA) platform, inspired by dot-blot technology, enables quantitative, cost-effective, high-throughput measurement of protein expression of total and phosphoproteins with high sensitivity and precision (Akbani *et al.*, 2014; Kornblau *et al.*, 2010; Liotta *et al.*, 2003; Uhlmann *et al.*, 2012). Initially presented in 2001, RPPA has been used in a variety of applications over the last two decades (Paweletz *et al.*, 2001; Spurrier *et al.*, 2008). The SuperCurve R package was developed to provide comprehensive modules for normalization, quality control, spatial adjustment and protein loading control in a single package (Hu *et al.*, 2007; Ju *et al.*, 2015; Kaushik *et al.*, 2014; Neeley *et al.*, 2012). Details of the Super curve modules are provided in Supplementary materials. To date, it has been used to process over 170 000 samples at MD Anderson Cancer Center and elsewhere. Some of the datasets generated using it, including 7578 samples from The Cancer Genome Atlas and 966 samples from the Cancer Cell Line Encyclopedia, can be found at The Cancer Proteome Atlas Portal, https://tcpaportal.org/ (Li *et al.*, 2013). Normacurve, another package based on SuperCurve, integrates simultaneous normalization (based on background fluorescence, spatial variation and variation in total spotted protein) with the quantitation (Troncale *et al.*, 2012). Another published modified version of SuperCurve assumes serial dilution of RPPA samples as a time-series structure and suggests taking the difference between the dilutions in order to minimize the influence of background noise (Sun *et al.*, 2015).

**Fig. 1.**
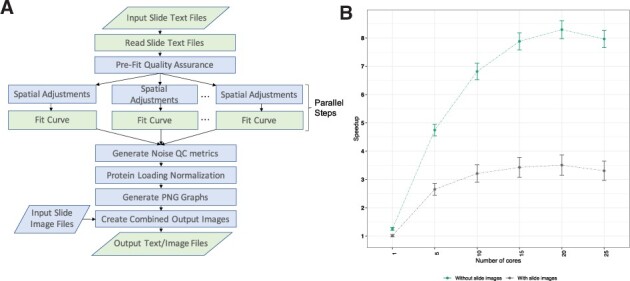
(**A**) *RPPA SPACE processing.* Processing steps executed by RPPA SPACE, showing serial and parallel steps. Steps in blue are optional. Parallelograms indicate input/output steps. (**B**) *Speedup of RPPA SPACE relative to its predecessor*, *SuperCurve.* For each run, 100 antibodies were randomly selected from a set of 250–500 antibodies. The means across 10 runs, each from a different set, are plotted here, along with standard error bars, as a function of the number of cores used. SuperCurve allows use of only one CPU core (A color version of this figure appears in the online version of this article.)

Here, we present RPPA SPACE (RPPA *S*uperposition *A*nalysis and *C*oncentration *E*valuation), a standalone R package, the successor to SuperCurve. Figure 1A shows the different steps implemented in RPPA SPACE to normalize raw data. Detailed guidelines on input and output formats are provided in the Supplementary material. The standalone R package offers substantial improvements over SuperCurve (Supplementary Table S1), as highlighted below.

## 2 Materials and methods

We obtained text files and slide images for 10 sets from the RPPA Functional Proteomics Core facility at MD Anderson Cancer Center. Experimental details of slide processing and quantitation are described at https://www.mdanderson.org/research/research-resources/core-facilities/functional-proteomics-rppa-core.html. The RPPA SPACE package (with different numbers of compute cores) and the SuperCurve package were run on all 10 sets individually by randomly selecting 100 antibodies from each set. RPPA SPACE analysis was repeated to study the effect of the number of antibodies by randomly selecting 20, 40, 60, 80 and 100 antibodies. Speedup for each set was calculated by the following formula:

*Speedup = Time required for SuperCurve run/Time required for RPPA SPACE run using specified number of cores*

The mean of the 10 runs was used to compute a single-point estimate shown in Figure 1B, and the standard deviation was used to compute the error bars.

## 3 Distinguishing features of RPPA SPACE

### 3.1 Improved quality control

1. Exclusion of poor-quality samples: Like SuperCurve, RPPA SPACE uses all spots from all samples on a slide to construct a single curve of intensity versus relative concentration that best fits all of the data. If there are a few outlier spots, their effect on the final curve is usually small. However, in some cases, a small number of large residuals can have a substantial effect. To mitigate such effects, RPPA SPACE allows users to specify which samples to exclude from all slides when creating the curves. Once the curves are created, the user can choose to use them to normalize data from the outlier samples. The suggested workflow is to run all samples and examine the output plots to determine which samples are outliers with large residuals, then choose which, if any, spots to exclude.
2. Noise estimation: It is crucial for a user to know the estimated level of random errors, or noise, in the measurements. Those parameters had thus far been difficult to quantitate for RPPA because they varied from one antibody to the next, and even from one slide to the next for the same antibody. To overcome that problem, we developed a simple, yet effective method to compute noise on a per-slide basis. The user runs several technical replicates on the slide that express the protein of interest. RPPA SPACE computes their standard deviation to estimate the magnitude of noise on the slide. Since they are technical replicates, any variations in their measurements cannot be attributed to biology and are concluded to be due to measurement errors. We found that the errors had a very low correlation with the magnitude of protein expression (*R* = 0.16 across 4261 slides, Supplementary Fig. S1). That implies that they can be used to estimate noise level across an entire slide because they don’t vary much based on the expression levels of individual samples. A high noise level (compared with prior data) indicates a poor-quality slide. RPPA SPACE also outputs the mean expression across the technical replicates. Like SuperCurve, RPPA SPACE allows for spatial adjustment using technical replicates (Neeley *et al.*, 2012), and the same replicates can be used for noise estimation. It is worth mentioning that, although RPPA SPACE algorithms have the potential to expand to other non-RPPA technologies in the future, in its current form, RPPA SPACE cannot be applied to one-dot format microarrays due to its requirement for serial dilutions.

### 3.2 Simpler requirements and improved flexibility

1. No slide design file required: The layout of an RPPA slide can vary among different laboratories from a single rectangular grid to multiple grids and sub-grids. SuperCurve requires users to specify the layout in a separate slide-design file. RPPA SPACE does not require that additional file. Instead, it automatically determines the slide design features (e.g. slide layout, dilution factor, series ID and spot type) from the first valid input file and assumes the same design for all subsequent slides in the set. A typical slide design handled by RPPA SPACE is described in Supplementary material.
2. Reduced dependencies and more flexible image handling: Unlike SuperCurve, RPPA SPACE does not require installing third-party software, i.e. ‘ImageMagick’ for image handling. RPPA SPACE can input image files in various formats (tiff, png, bmp, gif, jpg) and different orientations. The package has built-in functionality that enables the user to rotate the images for all the slides by multiples of 90° until they are properly oriented. The slide images are used in generating the final output image file, but they are not used in the quantitation. Conversion of the data from scanned image to numerical values is done outside of RPPA SPACE, so the image format (and its associated dynamic range) used for scanning is not part of the program.
3. Flexibility to use third-party protein loading correction: RPPA SPACE implements all the post-processing protein loading normalization methods available in SuperCurve, but unlike SuperCurve, it also allows the user to select ‘no normalization’, so protein loading adjustments can be done after RPPA SPACE using any user-given method.

### 3.3 Faster run time

SuperCurve typically takes 9–10 h to run a batch of 1056 samples and 500 antibodies in our laboratory (single core used, Intel Xeon CPU E7-4850, 2 GHz). RPPA SPACE has a much faster run time, in part due to the ability to use parallelization on multiple CPU cores for computationally expensive steps such as curve fitting and spatial correction (Fig. 1B). SuperCurve allows the use of only a single core. Another advantage in terms of speed is due to image processing: SuperCurve generates a computationally expensive and somewhat unnecessary image of curve estimations and residual plots along with the input slide image. In RPPA SPACE, image generation is optional. Figure 1B displays the speedup of RPPA SPACE over SuperCurve on batches of 1056 samples and 100 antibodies. By using 20 cores, we obtained a maximum speed up of 3.5-fold over SuperCurve with image generation on and 8.3-fold with it off. Interestingly, the performance appeared to degrade beyond 20 cores for our specific experimental setup, likely because the cost of integrating data from additional cores became higher than the benefit from adding them. We recommend an initial exploratory run to determine the appropriate number of cores to use for each experimental setup (e.g. 10–15 cores were appropriate for our application). Not surprisingly, run time increases linearly with an increasing number of antibodies in a batch, since each additional antibody just adds another sequential step to the process (Supplementary Fig. S2). A typical RPPA set requires two or three iterations of runs. The first run is used to detect any problematic slides, which are then corrected, if possible (e.g. by re-staining) and re-run. Two or three runs of SuperCurve at 9–10 h each translates into 2–3 additional business days of turnaround time. However, using RPPA SPACE a user can finish such a run in just over an hour, hence easily enabling the completion of two or three runs in a single business day.

### 3.4 Improved error reporting

Run-time errors are handled more effectively by RPPA SPACE; one problematic slide does not halt the processing of subsequent slides. Additionally, run-time error messages are more specific, and the fact that they are tracked in error text files and warning text files facilitates troubleshooting.

## 4 Conclusion

RPPA SPACE offers major improvements over its predecessor, SuperCurve (Supplementary Table S1), and its variants (Sun *et al.*, 2015; Troncale *et al.*, 2012). The variants of SuperCurve offer modified algorithms for data normalization and quantification, but they do not offer the advanced features of RPPA SPACE, such as enhanced quality control of the data, more flexible input requirements and the ability to utilize multiple CPU cores. RPPA SPACE runs up to eight times faster than SuperCurve (using multiple CPU cores) and has improved tolerance towards errors. It is currently being used as a replacement for SuperCurve by MD Anderson Cancer Center’s RPPA core facility, and it has been extensively tested on over 10 000 customer samples. We expect it to be useful for other researchers as well for normalizing raw RPPA data.

## Funding

This work was supported by National Institutes of Health/National Cancer Institute [CA210950 and CA264006] for Center for Cancer Genomics’ MD Anderson Genome Data Analysis Center, [CA16672] for the University of Texas MD Anderson Bioinformatics Shared Resource, [R50CA221675] for the MD Anderson RPPA Core Facility; Cancer Prevention and Research Institute of Texas (CPRIT) [RP210042 and RP160015]; and kind gift from the Miriam and Sheldon Adelson Research Foundation.

*Conflict of Interest*: R.A. is a consultant for the University of Houston. G.B.M. is a SAB/consultant for AstraZeneca, Chrysallis Biotechnology, Ellipses Pharma, ImmunoMET, Infinity, Ionis, Lilly, Medacorp, Nanostring, PDX Pharmaceuticals, Signalchem Lifesciences, Tarveda, Turbine, Zentalis Pharmaceuticals. G.B.M. has Stock/Options/Financial interests in Catena Pharmaceuticals, ImmunoMet, SignalChem, Tarveda and Turbine. G.B.M. licensed Technology HRD assay to Myriad Genetics and has DSP patents with Nanostring.

## Supplementary Material

btac665_Supplementary_Data

## Data Availability

The data used in this article will be shared on reasonable request to the corresponding author.
