# Supplementary material for: RPPA SPACE: an R package for normalization and quantitation of Reverse-Phase Protein Array data
Source: Bioinformatics. 2022 Oct 7;38(22):5131–3. doi: 10.1093/bioinformatics/btac665 (PMC9665860; doi:10.1093/bioinformatics/btac665)
Supplement: btac665_Supplementary_Data [file btac665_supplementary_data.zip › btac665_Supplementary_Data/Supplementary materials_Accepted.docx]

**Supplementary Material**

**RPPA SPACE: An R package for normalization and quantitation of Reverse-Phase Protein Array (RPPA) data**

Huma Shehwana^1^, Shwetha V. Kumar^1^, James M. Melott^1^, Mary A. Rohrdanz^1^, Chris Wakefield^1^, Zhenlin Ju^1^, Doris R. Siwak^2^, Yiling Lu^2^, Bradley M. Broom^1^, John N. Weinstein^1,3^, Gordon B. Mills^4^, Rehan Akbani^1*^

^1^Department of Bioinformatics and Computational Biology, The University of Texas MD Anderson Cancer Center, Houston, TX 77030, USA

^2^Department of Genomic Medicine, The University of Texas MD Anderson Cancer Center, Houston, TX 77030, USA

^3^Department of Systems Biology, The University of Texas MD Anderson Cancer Center, Houston, TX 77030, USA

^4^Knight Cancer Institute, Oregon Health and Science Center, Portland, OR 97210, USA

^*^To whom correspondence should be addressed

**1.1 RPPA data processing in SuperCurve R package**

The SuperCurve R package was first presented in 2007 (Ju, et al., 2015) as a modular software to analyze raw intensities of RPPA slides. Raw RPPA slide images are first processed using specialized software Array-Pro Analyzer (APA) or MicroVigene to quantify the mean intensity of each spot, background intensity, net spot intensity (mean intensity of the spot minus the mean background intensity), and position of each spot on the slide. The mean net spot intensity values from each slide can then be processed in the SuperCurve R package using three different modules. The first module focuses on measuring relative protein concentration from spot values of a given dilution series corresponding to each sample. The estimation of the relative concentration is based on the knowledge that for a given 5-step dilution series, each successive dilution will be 2 times more concentrated than the previous dilution (Hu, et al., 2007). SuperCurve constructs a curve between spot intensities on the y-axis and the relative concentration of each spot (x-axis). First, this curve is plotted for each individual dilution series, and as a second step, curves from all samples are superimposed to obtain a single unified curve, which is the main strength of the SuperCurve approach. Although users can choose parametric or non-parametric models to fit this curve, we recommend a non-parametric model fitting. By default, SuperCurve package outputs relative protein concentration but the user can also obtain absolute values.

As a second module, the SuperCurve package presented a quality control metric to differentiate between good and bad slides on the basis of probability scores ranging from 0 to 1. The classifier integrated multiple quantitative features (including signal intensity, background to signal ratio, coefficient of variation of each dilution step from all positive control samples, and dynamic range between different dilution steps of positive control samples) in a novel classifier using a generalized linear model and logistic function (Ju, et al., 2015).

Later, the third module of spatial correction was also added to the SuperCurve package (Kaushik, et al., 2014). We recommend printing multiple positive control samples and spreading them throughout the slide. Under ideal conditions, all of the positive control samples should show the same expression irrespective of their position on the slide and any difference in the expression will be attributed to technical or spatial artifacts. SuperCurve has a module to estimate these spatial effects and offset all the values in the respective grid such that the intensity of all positive control samples can be made equal to one another. In addition to these three modules, SuperCurve also allows users to normalize the RPPA data using different methods (Median polish, median, housekeeping, and variable slope method) (Ju, et al., 2015).

**1.2. Table S1: Comparison of SuperCurve and RPPA SPACE features**

| **Features** | **SuperCurve** | **RPPA SPACE** |
| --- | --- | --- |
| **Modules** | Four main modules are 1) measuring relative concentration 2) Spatial adjustment 3) quality control and 4) normalization | Four main modules 1) measuring relative concentration 2) Spatial adjustment 3) quality control and 4) normalization |
| **Quality control scores** | One quality control metrics based on four quantitative features of positive control samples (explained in section 1.1). | Two quality control metrics   1. Quality control score based on four quantitative features of positive control samples (explained in section 1.1). 2. Noise calculation: Standard deviation between positive control samples. |
| **Inclusion/exclusion of samples** | All samples on a slide should be used for curve fitting and normalization | Users can choose to exclude outlier samples from curve fitting and normalization |
| **Input requirement** | SuperCurve needs 1) slide images and 2) text files with intensity values of each slide in the dataset along with 3) one design file. | RPPA SPACE needs 1) slide images and 2) text files with intensity values of each slide in the dataset with the slide design specified. No separate design file needed. |
| **Slide images handling** | SuperCurve needs 'ImageMagick' software to be installed for image handling and proper orientation. | RPPA SPACE does not need any third-party software to be installed for image handling. |
| **Normalization** | SuperCurve does not output data without normalization. | RPPA SPACE allow user to output data with or without any normalization method depending on user specification. |
| **Run time** | - SuperCurve only uses one thread to process slide data. - SuperCurve needs slide images as an input to integrate it in output graph. | - RPPA SPACE benefits from parallel computing to enable faster run. - RPPA SPACE allow users to avoid time expensive step of integrating slide image to output plot. |
| **Error reporting** | Error in one slide halts the execution of entire dataset. | RPPA SPACE omits the execution of problematic slide and does not halt the execution of entire dataset. RPPA SPACE saves all errors in error log file. |

**1.3. Guideline to input and output files of RPPA SPACE**

**RPPA SPACE standard slide input format**

Like SuperCurve, RPPA SPACE accepts quantification data, and hence, slide images are first quantified using image quantification software e.g. ArrayPro or MicroVigene. RPPA SPACE expects all slide data inputs to be in a standard format with the first few columns specifying the design of the slide. To achieve that, the RPPA SPACE package includes one example of an R script along with the example data file that can help users to modify the script according to their own input. Slide images can also be optionally input as one image per slide (recommended) in different formats including tif, png, bmp, gif and jpg. Input slide images are only appended to the graphs if the user selects an option for creating combined output images.

Tab-delimited quantification file from each slide image should contain quantification data of one slide in one file and all slide files should be in the same folder. All quantification files should follow the same standard layout with a single row for each spot on the slide grid with the following columns.

- Order of the points in the file
- Main grid row number
- Main grid column number
- Subgrid column number
- Series ID for sample dilution series printed on the slide (0 for blank spot and negative controls)
- Spot type with possible values including “Sample”, “Blank”, “Buffer”, “NegCtrl”, “PosCtrl”, “PosCtrl-Noise”, and “Noise”.
- The decimal value of the dilution. A common 2-fold dilution series can be represented as 100, 50, 25, 12.5, 6.25, 3.125, and 1.5625.
- The net value of each spot value (Background value – Raw value).
- Background value around each spot.
- The horizontal position of the center of the spot.
- The vertical position of the center of the spot.
- The original order of each spot in the source file before converting to the standard format.

An example of a slide image and quantification file along with a conversion script (to a standard format) are available at [https://github.com/MD-Anderson-Bioinformatics/RPPA SPACE/tree/main/RPPA SPACE/inst/extdata](https://github.com/MD-Anderson-Bioinformatics/rppaspace/tree/main/RPPASPACE/inst/extdata).

**RPPA SPACE data processing steps**

RPPA SPACE package processes all the slides in the datasets in the following steps

1 – Read the quantification files from all the slides in the given directory.

2 – Perform quality control checks.

3 – Perform spatial adjustments on each slide.

4 – Perform curve fitting to estimate relative concentrations.

5 – Perform noise quality control measures.

6 – Generate graphical plots and save them in the output format.

7 – Perform selected normalization on the dataset.

8 – Write results in the output folder.

**Output files**

Two graphical png files are generated during RPPA SPACE execution with two respective plots in each png file. The first file shows input intensities vs calculated log2 concentration and a green line indicating the fitted curve. The second graph plot shows computed measures like raw residuals from the fit. The second graphical output file includes 1) a plot of residual vs estimated concentration and 2) a plot of the difference between sequential dilutions in the same dilution vs the average of these adjacent spots. Another, third combined output plot is generated if requested by users. This integrated output file contains all four plots from the previously described two output files along with an input slide image. In addition to graphical output, RPPA SPACE outputs processed data files with normalized data if the user selected any normalization method. Quality control results are also written to corresponding output files in csv format.

**Noise estimation**

It is recommended to include positive and negative control samples on the RPPA slide for various processes including spatial correction, curve estimation, and quality control metrics. In addition, some of the positive control samples can alternatively be used for noise estimation, a newly introduced quality control metric in RPPA SPACE package. As discussed in the previous section, spot.Type field of RPPA SPACE standard input can be used to specify PosCtrl, PosCtrl-Noise, or Noise. Depending on the designated value, these technical replicates will be differently treated as described below:

**Spot.Type = PosCtrl**

Samples labeled as PosCtrl will be used to calculate the Prefit quality control metric, estimate spatial correction and apply spatial correction results to these samples. Samples labeled as PosCtrl will neither be used to estimate the curve nor be fit to the curve. These samples will also not be used in the calculation of the noise quality control metric.

**Spot.Type = PosCtrl-Noise**

Like PosCtrl, PosCtrl-Noise samples will be used in pre-fit quality control metric calculation and spatial correction but will not be employed to calculate the curve for the slide. However, unlike PosCtrl samples, PosCtrl-Noise samples will be fit to the final estimated curve of the slide and will be used in the noise quality control metric.

**Spot.Type = Noise**

Noise samples will also be used for pre-fit quality control metrics. However, unlike PosCtrl or PosCtrl-Noise samples, noise samples will not be used to calculate spatial correction factors but calculated spatial correction factors will be applied to these samples prior to noise estimation. Noise samples will not be used to estimate the curve of the slide but will be fit to the curve. As the name indicates, noise samples will be used to estimate noise quality control.

**Noise calculation**

Samples labeled as PosCtrl-Noise and Noise will be directly used for Noise QC metric calculation. A slide K with a total of *k* PosCtrl-Noise and Noise samples (P) can be defined as

*K = {P_1_, P_2_, P_3_, ..., P_k_}*

Noise of each slide *(N_i_) = s(K)*

The final noise metric will be a vector of standard deviations with a single numerical value for each slide and the length of the vector will be equal to the number of slides in the dataset.

**Supplementary figures**

**Supplementary figure 1**

**
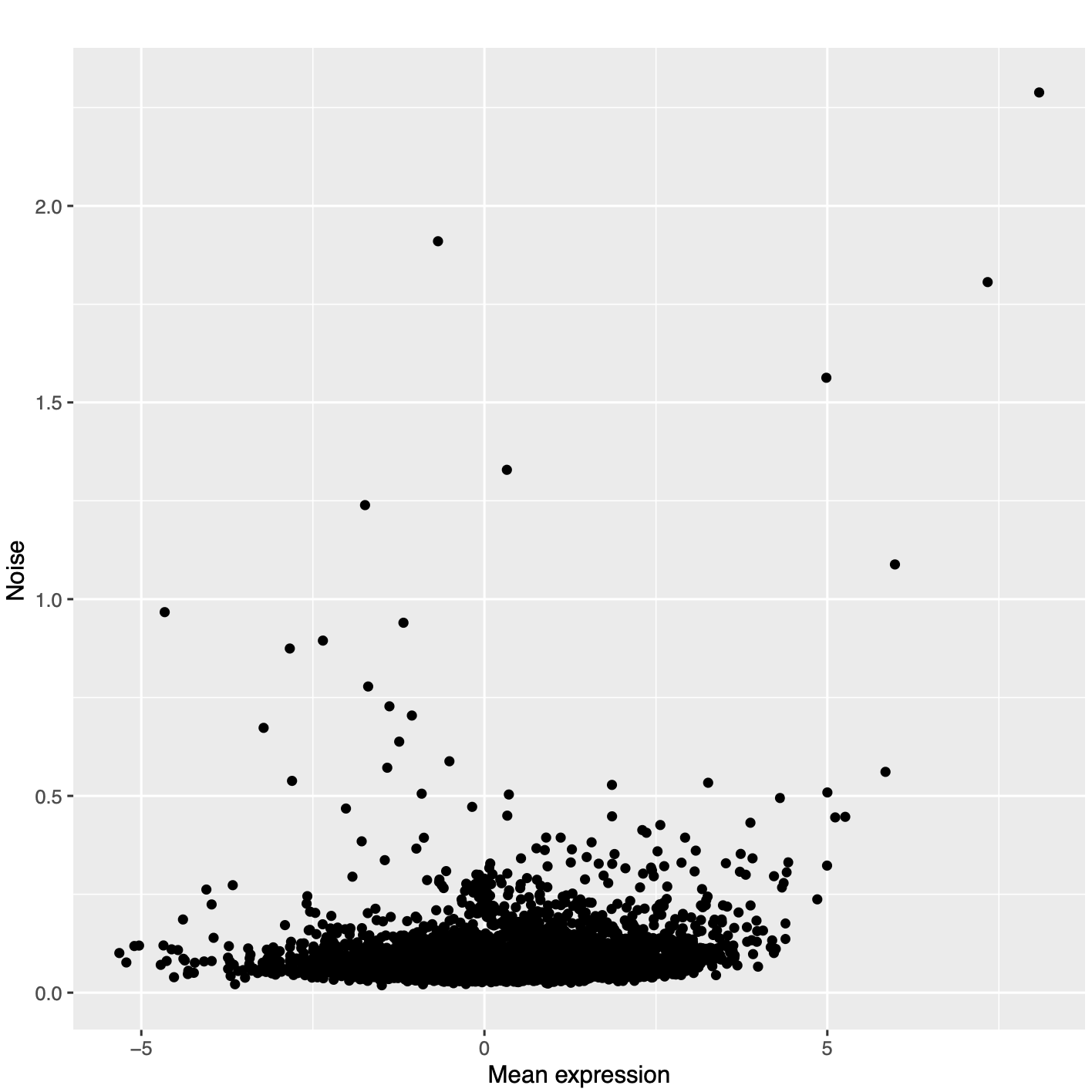
**

***Scatter plot of noise (y-axis) vs. mean expression (x-axis) of 4261 antibodies.*** Noise has a very low correlation with mean expression (R=0.16).

**Supplementary figure 2**

*
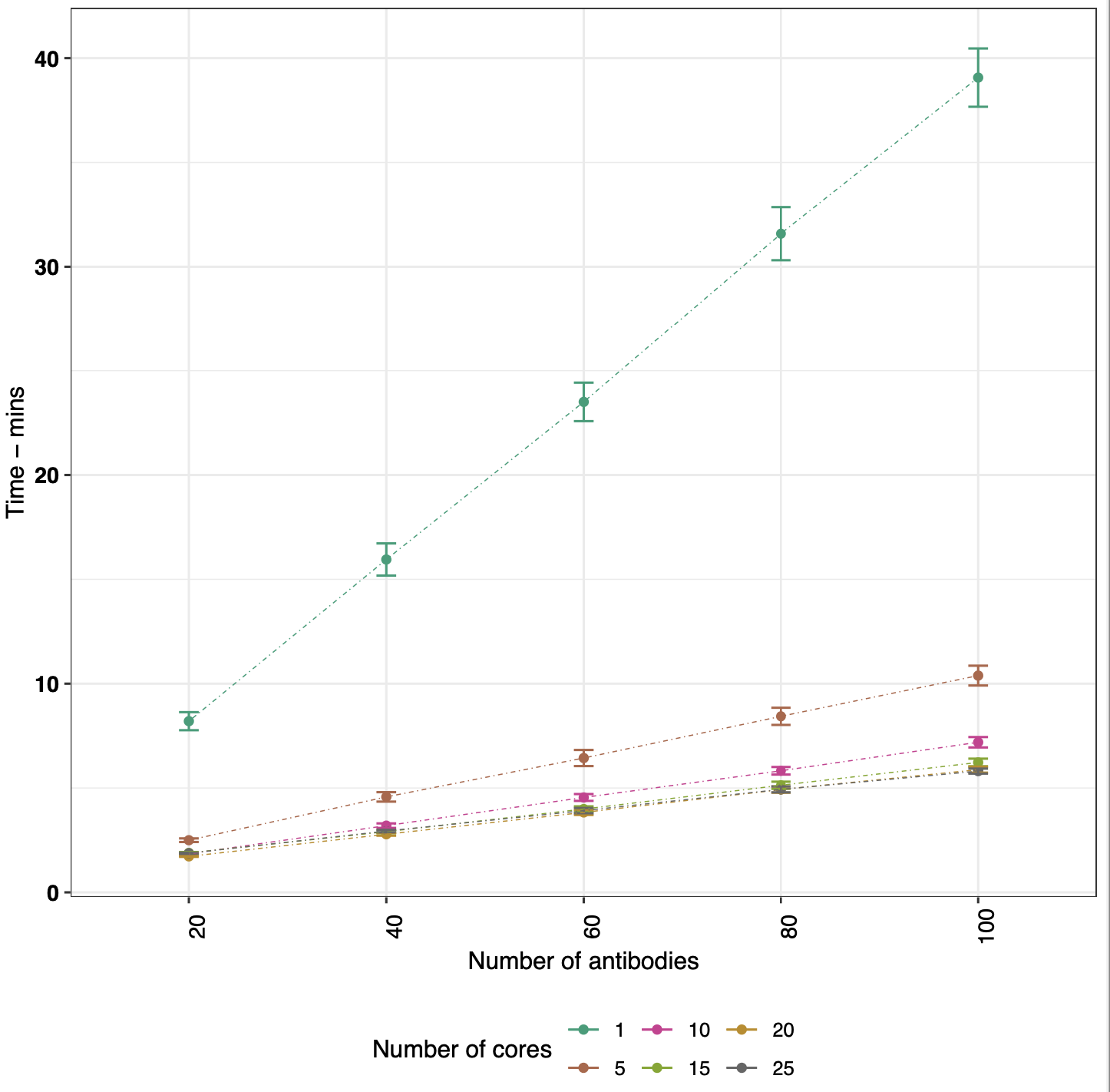
*

***Run time of RPPA SPACE vs. different numbers of antibodies and cores.*** RPPA SPACE was run ten times, each time on a different set, by selecting different numbers of antibodies and cores. Mean values across the ten runs are plotted, along with standard error bars.

**References**

Hu, J.*, et al.* Non-parametric quantification of protein lysate arrays. *Bioinformatics* 2007;23(15):1986-1994.

Ju, Z.*, et al.* Development of a robust classifier for quality control of reverse-phase protein arrays. *Bioinformatics* 2015;31(6):912-918.

Kaushik, P.*, et al.* Spatial normalization of reverse phase protein array data. *PLoS One* 2014;9(12):e97213.
